# Staged magnetic resonance-guided focused ultrasound thalamotomy for the treatment of bilateral essential tremor and Parkinson’s disease related tremor: a systematic review and critical appraisal of current knowledge

**DOI:** 10.3389/fneur.2024.1409727

**Published:** 2024-06-20

**Authors:** Simone Cesarano, Gennaro Saporito, Patrizia Sucapane, Federico Bruno, Alessia Catalucci, Maria Letizia Pistoia, Alessandra Splendiani, Alessandro Ricci, Ernesto Di Cesare, Rocco Totaro, Francesca Pistoia

**Affiliations:** ^1^Department of Biotechnological and Applied Clinical Sciences, University of L’Aquila, L’Aquila, Italy; ^2^Department of Neurology, San Salvatore Hospital, L’Aquila, Italy; ^3^Department of Radiology, San Salvatore Hospital, L'Aquila, Italy; ^4^Department of Neurosurgery, San Salvatore Hospital, L’Aquila, Italy

**Keywords:** MRgFUS, tremor, essential tremor, Parkinson’s disease, focused ultrasound, thalamotomy

## Abstract

**Introduction:**

Essential tremor (ET) and Parkinson’s Disease (PD) are debilitating neurodegenerative disorders characterized by tremor as a predominant symptom, significantly impacting patients’ quality of life. Magnetic Resonance-guided Focused Ultrasound (MRgFUS) Thalamotomy is an innovative therapeutic option for the treatment of unilateral medically refractory tremor with fewer adverse effects compared to traditional surgical interventions. A recent CE approval allows appropriate patients to have their second side treated.

**Objective:**

The objective of this systematic review was to analyze available current knowledge about the use of MRgFUS for the treatment of bilateral ET and PD related tremor, to identify the effectiveness and the risks associated with bilateral treatment.

**Methods:**

Eligible studies were identified by searching published studies in PubMed and Scopus databases from May 2014 to January 2024 and by identifying ongoing studies registered on the clinicaltrials.gov website. Data were summarized by considering the following information topics: the number of patients involved, the selected lesion target, the assessment tool used to evaluate clinical changes, the observed improvement, the reported side effects, and the time interval between the two treatments. The study was registered in PROSPERO (ID: CRD42024513178).

**Results:**

Nine studies were eligible for this review, 7 for ET and 2 for PD. The involved population included a variable number of patients, ranging from 1 to 11 subjects for ET and from 10 to 15 subjects for PD. The main lesional targets were the ventral intermediate nucleus of the thalamus, the pallidothalamic tract and the cerebellothalamic tract bilaterally. All studies investigated the tremor relief through the Clinical Rating Scale for Tremor (CRST) in patients with ET, and through the Unified Parkinson’s Disease Rating Scale (UPDRS) in patients with PD. A variable degree of improvement was observed, with all patients expressing overall satisfaction with the bilateral treatment. Adverse events were mild and transient, primarily involving gait disturbances, dysarthria, and ataxia. A standardized protocol for administering the two consecutive treatments was not identifiable; typically, the timing of the second treatment was delayed by at least 6 months.

**Conclusion:**

Available evidence supports the effectiveness and safety of staged bilateral MRgFUS treatments for ET and PD-related tremor.

## Introduction

Essential tremor (ET) and Parkinson’s Disease (PD) are two neurodegenerative disorders characterized by a symptomatic framework where tremor often assumes a predominant role (1). Tremor significantly impacts the patient’s quality of life, limiting autonomy and participation in social activities, and causing disability and social embarrassment (1, 2). Essential tremor is associated with a condition of slowly progressive action tremor, in the absence of other significant symptoms or a clear etiology, although recent evidence suggests, in some cases, an involvement of NOS3 or FUS genes mutations (3). It is a common disorder, affecting approximately 1% of the general population and 5% of those over 65 years of age. A family history is often reported, and a male predisposition to the development of ET is recognized in terms of both frequency and severity (4, 5). On the other hand, PD is a more complex disease, due to the degeneration of dopaminergic neurons within the nigrostriatal system. It is one of the most common causes of neurological disability, affecting approximately 1% of the population over 55 years of age, with a higher prevalence in males than females (6). Tremor may be variably combined with other symptoms such as bradykinesia and rigidity, so that different PD subtypes can be identified such as tremor-dominant PD, postural instability and gait disturbances (PIGD) PD and mixed forms (6). Both in ET and PD, pharmacological therapy has a poorly predictable effect on tremor. Moreover, the effect of pharmacological therapy tends to diminish as the disease progresses, especially in PD, following the end of the so-called honeymoon period during which the disease is still responsive to oral and transdermal therapy. As the disease progresses, motor fluctuations emerge, and tremor often become not satisfactorily controlled with oral medications. At that stage, advanced therapies should be offered to eligible patients. Traditional methods of surgical and radiotherapy treatment of tremor include deep brain stimulation (DBS), stereotactic radiosurgery (SRS) and radiofrequency thalamotomy (RF). These treatments target specific anatomical structures involved in motor control, primarily at the level of the thalamus which regulates the motor component through the pallidothalamo-cortical (extrapyramidal system) and cerebello-thalamo-cortical (muscle tone regulation) circuits. Specifically, the ventral intermediate nucleus (Vim) of the thalamus is the main target for ET patients and for some patient with tremor dominant PD. Although very effective, these procedures are associated with some risks related to surgical access and positioning of intracranial electrodes, particularly bleeding and infections (7). MRI-guided Focused Ultrasound (MRgFUS) is a new technology that enables non-invasive focal treatment within the brain, showing promising results (8–10). This technique uses a focused ultrasound beam that passes through the skull to reach the target area, without the need for anesthesia or craniotomy (11). Furthermore, the patient is awake and responsive throughout the whole procedure, allowing real-time assessment of any potential side effects (11). The same technique allows for the delivery of either low-intensity focused ultrasounds (LIFU) or high-intensity focused ultrasounds (HIFU) (12). The former may be used for transiently and non-invasively disrupting the blood brain barrier (BBB), allowing for localized delivery of drugs, genes, or other therapeutic agents. The latter are used for ablative purposes in the treatment of medically refractory tremor and neuropathic pain (13–17). During HIFU sessions, the patient’s head is shaved and fixed to a stereotactic frame, a flexible silicone membrane is applied to seal the space between the head and the transducer, and water at 15°C–20°C is used to reduce scalp overheating (13). Before the procedure, specific sequences of images are acquired, and the target to be treated is planned. Low-power sonifications are initially administered for 10–20 s to achieve a maximum temperature between 40°C and 42°C and assess the effect of sonification on the target. Once the choice of the target corresponds to the desired therapeutic effect without side effects, high-power sonifications (below 54°C) causing coagulative necrosis are administered (14). Possible complications include periprocedural transient symptoms that may occur during sonifications, such as headache, dizziness, vertigo, nausea, vomiting, scalp warmth sensation, and paraesthesia. These symptoms usually resolve within a few hours. In some cases, thalamotomy-related effects, due to the creation of a thalamic lesion, including gait disturbances or weakness of a limb, may persist longer, usually resolving within 3 months (17). Cognitive outcomes following unilateral MRgFUS thalamotomy have been also investigated, with findings indicating no deterioration, but rather a tendency toward slight improvement (18). The characteristics of this technique allow for immediate therapeutic effects and a rapid return to normal activities. Moreover, a significant advantage that has facilitated its rapid dissemination is the possibility of real-time feedback from the patient during the procedure, allowing for intraoperative evaluations (19).

The aim of this review was to systematically analyze the current knowledge about the use of MRgFUS for the bilateral treatment of ET and PD related tremor. The review focuses on the benefits and risks associated with bilateral procedures, the selection of anatomical therapeutic targets, and the strengths and limitations of the available studies in this field.

## Methods

This systematic review was performed according to the Preferred Reporting Items for Systematic Reviews and Meta-Analyses (PRISMA) guidelines, updated to 2020 (20). The study was registered in PROSPERO (ID: CRD42024513178) and the protocol can be found at the PROSPERO database. Data were analyzed and summarized using the Population, Intervention, Comparison, and Outcome (PICO) framework. The PICO question was as it follows: which is the efficacy and safety profile (outcome) of bilateral MRgFUS (intervention) in patients with ET and PD (population)? Comparison with unilateral MRgFUS was also reported where available. The research was conducted on PubMed and Scopus, by identifying articles indexed from May 2014 to January 2024. We conducted a search on both databases using the search terms “MRgFUS,” “focused ultrasound,” “thalamotomy,” combined with the terms “tremor,” “Parkinson’s disease” and “Essential tremor.” The search was restricted to humans and articles published in English. Only studies focusing on the bilateral application of MRgFUS have been considered. Specifically, original studies, including clinical trials, observational studies, case series and case reports addressing HIFU bilateral MRgFUS for ET or PD were eligible for this review. Studies lacking a clear definition of the study design and settings, letters, abstracts, studies not performed on humans, unpublished studies, and studies in which MRgFUS was used outside the neurological context were excluded. Duplicate publications were removed by manual check. The study selection process occurred through 2 phases: in the first phase, studies were selected through the reading of titles/abstracts, and in the second phase, through the reading of full texts. All articles were imported into an online software^1^ used during the screening process. The article selection process was carried out independently by two investigators (SC and GS) who initially assessed the study eligibility by screening titles and abstracts. Disagreements were resolved through discussion with a third investigator (FP). In the second phase, the full texts of the selected articles were evaluated. To also include ongoing studies, we conducted an additional search on the clinicaltrials.gov database from the beginning of indexing up to January 2024, using the same terms as those used for the database search. The included studies were individually described, and data were summarized based on the following main domains: the number of subjects treated bilaterally, the selected intracranial target, the interval between the two treatments, the clinical tool used to evaluate tremor, the degree of observed improvement, and the occurrence of adverse events. Additional information regarding patients’ satisfaction with the treatment, cognitive status, and quality of life was provided where available.

## Results

**Figure 1:**
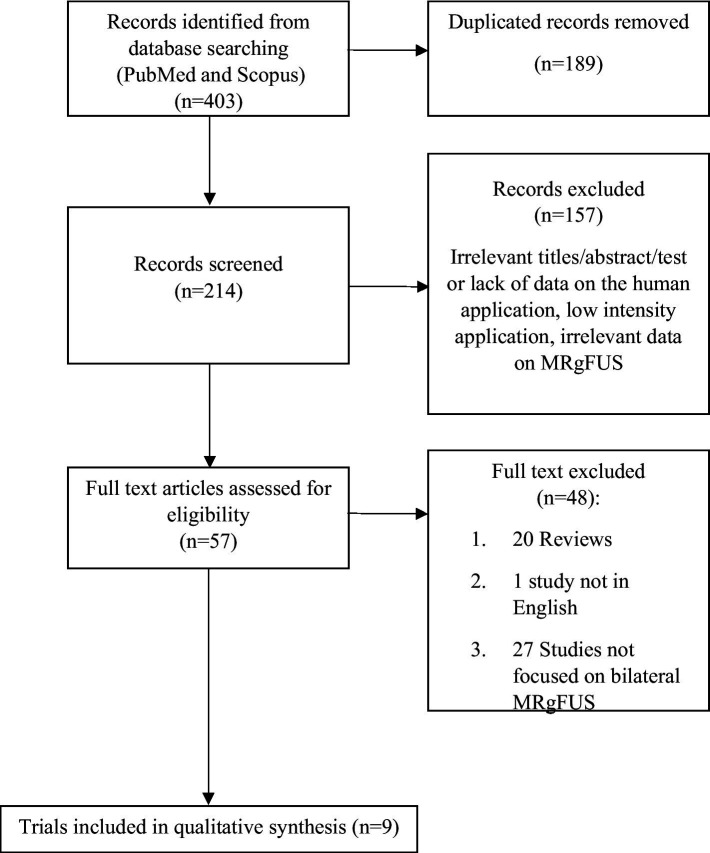
Flow diagrams of studies selected.

A total of 403 records were identified through the search on PubMed and Scopus. After removing duplicates, 214 articles underwent screening, with 57 considered relevant for full-text analysis. Forty-eight studies were subsequently excluded: 20 did not meet the eligibility criteria in terms of study design, 27 did not focus on bilateral MRgFUS application, and 1 was excluded because the full text was in a language other than English. Regarding ongoing studies, a search on clinicaltrial.gov identified 14 articles, none of which met the criteria for inclusion in this review as they did not focus on bilateral MRgFUS application. At the end of the selection process, 9 studies were considered eligible (Figure 1 and Tables 1, 2).

**Table 1:** Bilateral MRgFUS in ET.

Study	Design, country	Included subjects/number of treatments	Age range	Disease treated	Disease duration (year)	Target of MRgFUS	Interval between procedures	Temperature achieved	Outcomes	Assessment time points	Results	Adverse events
Gallay et al., 2016	Case series, Switzerland	21 (3 bilateral treatment)	69 ± 9.2	Essential bilateral tremor	29.9 ± 15	Cerebellothalamic tract	1 year	54–60°C	-ERTS (HF16; HF32) MoCA HADS	3 months; 1 year	Mean improvement HF16: Group 1: 40% Group 2: 90% Improvement patient treated bilaterally HF16: 75% e 88%, respectively. MoCA and HADS not significantly changed	Worsening of pre-existing gait instability in one patient at 1 year.
Ito et al., 2020	Case report, Japan	1	57	Essential bilateral tremor	1	Ventral intermediate thalamic nucleus (Vim)	8 months	Lefts side: 47.3 ± 6.9°C (range: 40–60°C) Right side: 48.2 ± 6.1°C (range: 43–59°C)	CRST EQ-5D-3L	1 month after second treatment	Tremor disappeared in both hands. EQ-5D-3L: 11112 after first procedure, and 11,111 after the second	none
Bruno et al., 2020	Case report, Italy	1	36	ET	8	Vim	24 months	Not reported	CRST QUEST MoCA	24 h; 1 month; 6 months, 1 year after right side treatment 24 h; 1 month; 6 months after the second treatment	Right side: CRST: 15, 68.7% reduced. QUEST: 7, 86% reduced. MoCA: 30, 11.11% improved. Left side: CRST: 18, 50% reduced. QUEST: 3, 70% reduced. MoCA: no difference	none
Iorio-Morin et al., 2021	Prospective single-arm, single-blinded trial, Canada	10	71.5 ± 7	Essential tremor	>3	Vim	Median 9 months (range, 7–56 months)	62°C (range, 59°C–63°C)	Quality of life and disability: QUEST EQ-5D-5L CRST	2 h; 1 month and 3 months	QUEST 15.4 ± 11.3 (95%; *p* = 0.004) CRST part c: 2.4 ± 2.2 (mean difference, 4.2; 95%; *p* = 0.005) EQ-5D-5L: 80.5 16.1 (mean difference, 6.9; 95%; *p* = 0.013)	dysphagia occurred in 2 patients, only reported the symptom after 3 months.3 of the 10 patients experienced a lasting grade 1 or 2 complication: 4 had transient events that recovered, and 3 did not experience any complication.
Martinez-Fernandez et al., 2021	Prospective case series, Spain and Switzerland	9	71 ± 6	ET	31 ± 15	Vim	24 ± 18 months	Not reported	Safety and efficacy: CRST Incidence and severity of treatment related complication	4 weeks ± 14 before second thalamotomy; 6 months ±30 after second thalamotomy	CRST: 15.5 ± 9.4 (71%; *p* < 0.001)	Five out of nine patients developed transient gait instability. One patient showed dysarthria, remitted in 4 weeks. Three patients developed mild perioral sensory disturbances
Fukutome et al., 2022	Retrospective observational, Japan	5	57.4 ± 17	ET	>6	Vim	27.8 ± 11.48 months	55°C	CRST and part C VAS MMSE FUJISHIMA GRADE	3 months after each operation	CRST: 21.8 (65.72% reduction) CRST part C: 2.6 (85.87% reduction) MMSE: 28–30 FUJISHIMA GRADE: 10 VAS: 74 ± 19.7	None after the first operation. After second operation 3 patient experienced permanent slight dysarthria and dysthesia of the complement.
Scantlebury et al., 2023	Prospective case series, Canada	11	71 ± 8	ET	33 ± 18	Vim	35.27 ± 23.59 months	Not reported	CRST part A-B FDA-2 QUEST Gait and Stance itemo from SARA	3–6 months	CRST: 8 ± 4.95; *p* < 0.001 QUEST improved in “financial,” *p* = 0.0043; “Hobbies,” *p* = 0.007; “physical,” *p* = 0.001; “psychosocial” *p* = 0.002	Short lasting (<24 h) shoulder pain, nausea, headache

**Table 2:** Bilateral MRgFUS in PD.

Study	Design, country	Included subjects/number of treatments	Age range	Disease treated	Disease duration (year)	Target of MRgFUS	Interval between procedures	Temperature achieved	Outcomes	Assessment time points	Results	Adverse events
Gallay et al., 2020	Prospective case series, Switzerland	52 (15 bilateral treatment)	67 ± 10	Chronic therapy resistant PD	10 ± 5.3	Pallidothalamic tract	At least 6 months	43°, 240 CEM	Primary: UPDRS; GSRt; GSRb; reduction drug intake; off dystonia; on dyskinesias; sleep disturbances; pain; Secondary: MoCA; WHOQOL; HADS	2 days; 3 months; 1 year	At 1 year: UPDRS—UPDRS reduction: 46% on-medication (*n* = 21; *p* < 0.001) and 51% off-medication (*n* = 25; *p* < 0.001); GRSb: 69 ± 27 (*n* = 27, median: 80, 85% improved by ≥50%) GSRt 82 ± 22 (*n* = 29, median: 90, 93% improved by ≥50%). Secondary outcomes did not reached statistically significance differences	One patient with scalp hypoesthesia fully recovered in 3 months. One patient suffered from a short-lived intense anxio-depressive episode from which he rapidly and completely recovered. Seven patients reported increased (two patients) or new (five patients) speech difficulties (UPDRS II, item 5); at 1 year, they were 2 and 4, respectively.
Gallay et al., 2021	Retrospective observational, Switzerland	10	63 ± 5	Chronic therapy resistant PD	10.2 ± 4.6	Pallidothalamic tract	20 ± 10 months (median: 16.5; range: 5–38)	43240CEM	Primary: UPDRS-dyskinesias off- and on-dystonia drug intake Secondary: MoCA WHOQOL . HADS	before second side treatment; 1 year	UPDRS 29 ± 11 (52% reduction; *p* < 0.007) dyskinesias (choreoathetosis) were suppressed in four over four, dystonia in four over five patients 7/10 stopped al drug intake, alla stopped dopamine agonist. Secondary outcomes changes were not statistically significant.	One patient suffered hiccup and breathing difficulties, regressed in 10 months. One patient reported to be slightly dragged to his right side, improved after bilateral PTT. At 1 year after bilateral PTT, one patient experienced episodes of uncontrollable laughter.

Both for ET and PD data were summarized according to the PICO framework (Population of involved patients, Intervention, Comparison with unilateral treatment where available, Outcome identified by the efficacy and safety profile of the intervention). Individual studies were described in chronological order, according to the year published, from the oldest to the most recent ones.

### Bilateral MRgFUS in ET

Among the identified studies, 7 papers focused on the bilateral application of MRgFUS for the treatment of medically refractory ET.

In the prospective study by Gallay et al. (21), 21 patients treated with cerebellothalamic tractotomy (CTT) 5.0 mm posterior to the mid-commisural line (MCL) in the anteroposterior (AP) direction, 8.0 mm lateral to the thalamo-ventricular border in the mediolateral (ML) direction, and 3 mm below the intercommissural plane, were described: a subsample of patients (*n* = 3) underwent bilateral treatments, with a one-year interval between the two sessions. Tremor reduction was assessed through the Clinical Rating Scale for Tremor (CRST). Measures of tremor relief were not provided separately for patients undergoing unilateral and bilateral treatments, respectively: a 55% global CRST reduction was reported at the 1-year follow-up. Bilateral treatment did not produce side effects apart from a persistent slight worsening of pre-existent gait instability in a patient with concomitant documented polyneuropathy and cervical canal stenosis.

In the case-report by Ito et al. (22), a 57-year-old patient treated with bilateral thalamotomy was described. The selected intracranial target was the Ventral Intermediate (VIM) nucleus bilaterally, targeting a point 6.0 mm anterior to the posterior commissure (PC) and 14.5 mm left of the midline, and 1.5 mm above the anterior commissure (AC)-PC plane. The interval between the two treatments was 8 months. Tremor was assessed using the CRST, which revealed complete tremor remission in both hands (no specific percentages of reduction are reported). With respect to side effects, the patient reported dysesthesia in the right occipital region following the first treatment, which spontaneously disappeared 1 month later. No other adverse events occurred following the second treatment. The patient expressed global satisfaction with the treatment, as endorsed by the reported improvement of the EuroQOL (Quality of life) 5-dimension 3-level (EQ-5D-3L) score.

In the case-report by Bruno et al. (23), a 63-year-old patient with an 8-year history of essential tremor with progressive resistance to pharmacological therapy was described. The patient received a bilateral MRgFUS treatment. The selected intracranial target was the VIM bilaterally, 14 mm laterally from the AC-PC line, 6.7 mm anteriorly from the PC (halfway between 1/3 and 1/4 of the AC-PC distance), 1 mm above the AC-PC line. The time interval between the two treatments was 24 months. Tremor was assessed through the CRST, with scores significantly decreasing following both the procedures (from 48 to 23 for the right side at 6 months and from 36 to 18 for the left side at 6 months). No side effects were reported. Cognitive functions, as assessed through the Montreal Cognitive Assessment (MoCA) scale, remained unchanged following bilateral thalamotomy. The quality of life, investigated through the Quality of Life in Essential Tremor Questionnaire (QUEST), showed a 86% improvement after the treatment of the left VIM and a further 70% after the treatment of the right VIM.

In the prospective study by Iorio-Morin et al. (24), 10 patients receiving bilateral MRgFUS thalamotomy were described. The selected intracranial target was the Vim nucleus bilaterally: the coordinates were 15 mm lateral to the midline or 11 mm lateral to the wall of the third ventricle, 25 mm anterior to the PC along the intercommissural line between the AC and PC and 3 mm superior to the AC-PC plane. The median interval between the two treatments was 9 months. Overall tremor was assessed through the CRST, which revealed a relevant global improvement following the second procedure (38.1 ± 7.5) before the second procedure vs. 20.9 ± 6.4 after the second procedure (*p* < 0.0001) at the 3-month follow-up. Adverse events included transient limb ataxia, dizziness, or neglect, all of which fully resolved by the 3-month follow-up. However, two patients experienced persistent dysphagia at the 3-month follow-up. Quality of life, as assessed through the QUEST, significantly improved after the second-side thalamotomy (mean QUEST score decreased from 35.1 after the first procedure to 15.4, *p* = 0.004 at 3 months following the second procedure).

In the prospective study by Martinez-Fernàndez et al. (25), 9 patients receiving bilateral MRgFUS treatment were described. The selected intracranial target was the thalamus bilaterally and the mean interval between the two treatments was 24 months. Specific target coordinates are not reported in the study. Tremor was assessed through the CRST, showing improvement in all patients: the total CRST score decreased by 71% from baseline to the second procedure, with a 44% reduction after the first thalamotomy (*p* = 0.004) and an additional decrease of 50% after the second procedure (*p* = 0.008). Adverse events following the second treatment were mild and transient, including gait instability in five patients, worsening of pre-existing gait instability in one patient, dysarthria in one patient, and mild perioral sensory disturbances in two patients. All these symptoms showed full improvement within a few weeks after the last treatment. No differences after the second thalamotomy were observed in cognitive functions. In addition, the evaluation of voice through the Voice Handicap Index-30 (VHI) revealed a 40% improvement (25).

In the retrospective study by Fukutome et al. (26), 5 patients treated with bilateral VIM thalamotomy were described: the target was 11 mm lateral to the third ventricle wall, 5–5.5 mm posterior to the midcommissural point (MCP) at the level of the intercommissural line. The second procedure was performed slightly anteriorly and superiorly to the first, to avoid symmetrical lesioning. An average interval of 27.8 months between the two procedures was reported. Tremor reduction was assessed through the CRST. Significant improvement was observed after both the first and the second procedure, with the CRST score decreasing from 63.6 at baseline to 49.2 before the second intervention and to 21.8 after the second intervention (no significance level values were reported). No patients reported adverse events after the first sonification. However, following the second procedure, three patients experienced transient symptoms, including numbness of the lips, paresthesia, or limb weakness, lasting from a few days to 3 weeks. One patient reported permanent dysarthria and paraesthesia of the tongue. All patients expressed satisfaction with the treatment, as evidenced by an average visual analog scale (VAS, range 0–100) score of 74. Cognitive functions were assessed using the Mini-Mental State Examination (MMSE), with scores consistently maintained between 28 and 30 even after the second intervention. Quality of life, as assessed through part C of the CRST, improved by 85.87%, decreasing from a baseline value of 18.4 to 8.2 after the first procedure and to 2.6 following the second MRgFUS procedure (no significance level values were reported).

Finally, in the prospective study by Scantlebury et al. (27), 11 patients undergoing a second MRgFUS intervention were described. The selected intracranial target was the thalamus bilaterally and the mean interval between the two treatments was 35 months. Specific target coordinates are not reported in the study. Tremor severity was assessed using the CRST scale, which revealed a significant improvement after both the first and the second procedure: CRST scores for the targeted hand improved after each MRgFUS (*p* < 0.001), while the untargeted-hand tremor had no significant change. Many periprocedural adverse effects were detected, including transient shoulder pain in two patients, headaches in two patients, and nausea in one patient, all of which resolved quickly. Persistent perioral or finger paraesthesias were identified in four patients at the 6-month follow-up, although the symptoms did not impact their activities of daily living. Concurrent improvements in QUEST scores in the “financial,” “hobbies/leisure,” “physical,” and “psychosocial” domains were recognized.

### Bilateral MRgFUS in PD

In the prospective study by Gallay et al. (28), a series of 52 patients was described. Among them, 15 patients underwent bilateral MRgFUS, with two patients receiving the treatment in a single session and the others undergoing staged sessions. The selected intracranial target was the pallidothalamic tract 6.5 mm from the medial thalamic border and 1 mm posterior to the MCL (28). The time interval between the two procedures was at least 6 months. Tremor was evaluated using the Unified Parkinson’s Disease Rating Scale (UPDRS). Measures of tremor relief were not provided separately for patients undergoing unilateral and bilateral treatments, respectively: a 37% total UPDRS III score reduction was reported at 3 months (*p* < 0.01) and a 46% reduction at 1 year (*p* < 0.001). Irregular final follow-ups were obtained for the different patients: a small group of patients receiving bilateral treatment had a 1-year follow-up (only four patients), showing similar results compared to patients receiving unilateral treatment. A 55% reduction of the mean L-Dopa intake was also observed. No adverse effects related to the bilateral nature of the procedure were identified. Regarding the perception of quality of life, statistical significance was only reached for the World Health Organization Quality of Life (WHOQOL) item 2 (How satisfied are you with your health?) at 3 months (*p* < 0.001) and at 1 year (*p* < 0.005) and for the WHOQOL item 1 (how would you rate your quality of life) at 3 months (*p* = 0.002).

In a retrospective study by Gallay et al. (29), data confined to 10 patients with PD treated with bilateral MRgFUS are reported. The selected intracranial target was the pallidothalamic tract using the same targeting protocol as in the previous study (28). Tremor was assessed through the UPDRS, whose total score decreased from 65 ± 25 at baseline to 29 ± 11 1 year after the second intervention (52% reduction, *p* < 0.007). A reduction of the mean L-Dopa intake was also observed, from 690 ± 250 mg to 110 ± 190 mg 1 year after the second treatment. Seven patients had completely discontinued pharmacological therapy, and all 10 included patients had stopped using dopaminergic agonists. Regarding adverse events, one patient experienced respiratory and language difficulties, which resolved after 10 months. Another patient reported episode of falls and a deviation to the right while walking, with all symptoms resolving within 1 week. Cognitive effects were evaluated using the MoCA, but no cognitive changes were observed after the two procedures. The mean WHOQOL score showed a slight improvement, increasing from 93 ± 11 to 95 ± 11, but these changes were not statistically significant.

## Discussion

Our systematic review indicates that bilateral MRgFUS treatment could be an effective and promising clinical option for managing patients with ET and PD when tremor is refractory to pharmacological therapy. Available studies suggest that bilateral MRgFUS often yields better results compared to unilateral treatment, particularly in terms of improvements in quality of life, with an additional positive effect being observed after the second procedure (22, 24, 26, 28). The time elapsed between the first and the second intervention can vary from 6 months to 3 years, with significant heterogeneity observed across the studies. The limb initially treated typically continues to benefit from the effects of the first procedure, and no severe or persistent adverse effects have been observed following the treatment of the second limb. It can be hypothesized that postponing the second procedure ensures that the circuits involved, which were modulated during the first lesion, undergo stabilization before proceeding with contralateral treatment. This is contrary to what occurs with the MRgFUS management of refractory pain, where bilateral lesioning is performed in a single session. The difference is likely attributable to the fact that both ET and PD, unlike pain syndromes, are neurodegenerative diseases with a progressive and somewhat unpredictable natural history: thus, staging the second procedure may be preferable and more cautious. Staging the second treatment allows employing a waiting period to monitor the stability of the effects achieved with the initial procedure and to evaluate potential responses, including adaptive or maladaptive changes, in the broader neuronal circuits passing through the lesion site but extending beyond it (23, 29). The selected intracranial target is almost always the VIM. However, Gallay et al. (28) suggested the utility of targeting the pallidothalamic tract, particularly in bilateral procedures, to preserve the integrity of the thalamocortical network, thereby reducing motor and cognitive adverse events: however, their findings are too much preliminary and heterogenous to draw conclusions and deserve further investigations through specifically designed longitudinal case–control studies.

Overall, patient’s satisfaction following the second procedure and the safety profile are high, due to the minimally invasive nature of the technique and the rapid recovery of autonomy in activities of daily living. Given the rapid increase in the incidence of neurodegenerative diseases (30), ensuring a bilateral solution to the problem of medically refractory tremor significantly improves the quality of life of affected patients. The reduction in tremor, along with the preservation of all neurological functions, including cognitive functions, is an important objective in managing patients with ET or tremor-dominant PD. In the latter, of course, other motor symptoms and signs of the disease such as bradykinesia and rigidity are not influenced by MRgFUS, even some improvements following the second procedure have been reported (28): however, whether these improvements have been causally related to the procedure is yet to be determined. Nevertheless, the reduction in tremor alone can positively impact patients’ autonomy and quality of life. Some studies addressing unilateral MRgFUS thalamotomy confirmed the safety of the MRgFUS approach even with respect to cognitive functions (18). The contribution of these studies in the assessment of cognitive functions after unilateral MRgFUS was important in planning bilateral procedures. Indeed, while any adverse effects related to motor or sensory functions, language, or coordination can be directly ruled out during intraoperative monitoring, the same cannot be done for cognitive adverse events, which may manifest weeks or even months after the procedure. The data obtained in this context are highly reassuring because they not only indicate the absence of cognitive complications, even in the long term, but also suggest that there may be a slight improvement in overall cognitive performances of patients managed with unilateral MRgFUS thalamotomy. This improvement is likely driven by a reduction in patient distractibility due to the tremor itself, a reduction in social embarrassment caused by the tremor and an improvement in anxiety levels. The studies included in this systematic review confirm this aspect, regardless of the site of sonification treatment; in fact, the cognitive performance of the patients examined has never been affected, and in most cases, it has even been enhanced. Regarding adverse effects, the most frequent ones were dysarthria, ataxia, and gait disturbance. However, both the percentage and severity of adverse effects are progressively decreasing. This trend can be attributed to improved localization techniques, which enhance precision, and a more accurate differentiation from pre-existing symptoms related to the disease, thereby excluding them from consideration as adverse events (31). Given the heterogeneity of intervention protocols in various studies, it was not possible to highlight an optimal protocol for the bilateral administration of MRgFUS. However, all studies were based on the evaluation of functional and cognitive outcomes of the first treatment before deciding to proceed with contralateral sonification. Therefore, there is still no strong recommendation for the use of bilateral thalamotomy to date: attention must be paid to patient selection criteria for this procedure, preferably identifying those who have not experienced adverse effects after the first sonification session and who have maintained good neuropsychological performance in the following months (26).

In conclusion, this systematic review suggests that bilateral thalamotomy treatment with MRgFUS represents an effective and safe alternative approach in the treatment of patients diagnosed with medically refractory ET or PD related tremor. The data were collected in a limited and heterogeneous sample, but they are promising and encouraging. Overall, the studies are too heterogeneous to allow for a robust comparison of the data and generalization of the results to all patients with ET and PD. High-quality evidence is crucial to reach higher levels of evidence and standardizing the protocols used.

## Data availability statement

The original contributions presented in the study are included in the article/supplementary material, further inquiries can be directed to the corresponding author.

## Author contributions

SC: Writing – original draft, Data curation, Formal analysis, Investigation, Methodology. GS: Conceptualization, Data curation, Formal analysis, Writing – original draft. PS: Formal analysis, Investigation, Writing – review & editing. FB: Conceptualization, Writing – review & editing. AC: Conceptualization, Writing – review & editing. MP: Conceptualization, Writing – review & editing. AS: Conceptualization, Writing – review & editing. AR: Conceptualization, Writing – review & editing. EC: Conceptualization, Writing – review & editing. RT: Conceptualization, Writing – review & editing. FP: Conceptualization, Data curation, Formal analysis, Investigation, Methodology, Writing – review & editing.

## Footnotes

^1^ www.rayyan.ai
